# Byakangelicol suppresses TiPs-stimulated osteoclastogenesis and bone destruction via COX-2/NF-κB signaling pathway

**DOI:** 10.1093/rb/rbad092

**Published:** 2023-10-25

**Authors:** Zhidong Wang, Huaqiang Tao, Miao Chu, Lei Yu, Peng Yang, Qiufei Wang, Jun Lu, Huilin Yang, Zhenheng Wang, Hailin Zhang, Dechun Geng

**Affiliations:** Department of Orthopedics, The First Affiliated Hospital of Soochow University, No. 188 Shizi Street, Suzhou 215000, China; Department of Orthopedics, The First Affiliated Hospital of Soochow University, No. 188 Shizi Street, Suzhou 215000, China; Department of Orthopedics, The First Affiliated Hospital of Soochow University, No. 188 Shizi Street, Suzhou 215000, China; Department of Orthopedics, The First Affiliated Hospital of Soochow University, No. 188 Shizi Street, Suzhou 215000, China; Department of Orthopedics, The First Affiliated Hospital of Soochow University, No. 188 Shizi Street, Suzhou 215000, China; Department of Orthopedics, Changshu Hospital Affiliated to Soochow University, First People’s Hospital of Changshu City, Changshu 215500, China; Division of Sports Medicine and Adult Reconstructive Surgery, Department of Orthopedic Surgery, Nanjing Drum Tower Hospital, Affiliated Hospital of Medical School, Nanjing University, 321 Zhong Shan Road, Nanjing 210000, China; Department of Orthopedics, The First Affiliated Hospital of Soochow University, No. 188 Shizi Street, Suzhou 215000, China; Department of Orthopedics, The First Affiliated Hospital of Soochow University, No. 188 Shizi Street, Suzhou 215000, China; Department of Orthopedics, Jiangyin People’s Hospital Affiliated to Nantong University, No. 163 Shoushan Road, Jiangyin 214400, China; Department of Orthopedics, The First Affiliated Hospital of Soochow University, No. 188 Shizi Street, Suzhou 215000, China

**Keywords:** COX-2, TiPs, inflammation, byakangelicol, NF-κB

## Abstract

Aseptic loosening (AL) is considered a significant cause of prosthesis revision after arthroplasty and a crucial factor in the longevity of an artificial joint prosthesis. The development of AL is primarily attributed to a series of biological reactions, such as peri-prosthetic osteolysis (PPO) induced by wear particles around the prosthesis. Chronic inflammation of the peri-prosthetic border tissue and hyperactivation of osteoclasts are key factors in this process, which are induced by metallic wear particles like Ti particles (TiPs). In our *in vitro* study, we observed that TiPs significantly enhanced the expression of inflammation-related genes, including COX-2, IL-1β and IL-6. Through screening a traditional Chinese medicine database, we identified byakangelicol, a traditional Chinese medicine molecule that targets COX-2. Our results demonstrated that byakangelicol effectively inhibited TiPs-stimulated osteoclast activation. Mechanistically, we found that byakangelicol suppressed the expression of COX-2 and related pro-inflammatory factors by modulating macrophage polarization status and NF-κB signaling pathway. The *in vivo* results also demonstrated that byakangelicol effectively inhibited the expression of inflammation-related factors, thereby significantly alleviating TiPs-induced cranial osteolysis. These findings suggested that byakangelicol could potentially be a promising therapeutic approach for preventing PPO.

## Graphical abstract

**Figure:**
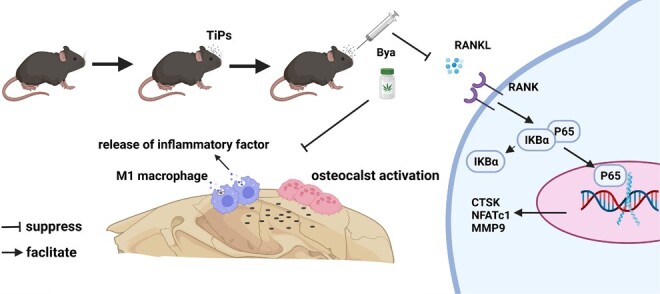

## Introduction

Total joint replacement (TJR) is a commonly used and effective treatment for patients with conditions like osteoarthritis, rheumatoid arthritis and femoral head necrosis, as well as other end-stage joint diseases [1, 2]. However, despite its effectiveness, TJR can be complicated by issues such as aseptic loosening (AL) and peri-prosthetic osteolysis (PPO) [3, 4]. These complications, which are triggered by inflammation, can lead to implant failure in up to 25% of recipients. Despite advancements in implant technology, surface treatments, biomaterials and targeted interventions, the issue of prosthetic loosening remains unresolved. Current treatment strategies lack effective methods to counteract inflammatory osteolysis, often resulting in the need for costly surgical revisions, shortened implant lifespan and compromised clinical outcomes [5–7]. Therefore, there is an urgent need to further investigate the pathogenic mechanisms that cause inflammatory osteolysis in prosthetic joint replacement and develop innovative therapeutic strategies to effectively tackle this significant challenge.

Macrophage polarization plays a critical role in regulating inflammation and is a key factor in immune responses and tissue homeostasis [8, 9]. In response to different environmental signals, macrophages can exhibit various functional phenotypes, which can be broadly categorized into two extremes: the pro-inflammatory M1 phenotype and the anti-inflammatory M2 phenotype [10, 11]. M1 macrophages, activated by pro-inflammatory stimuli, release cytokines such as TNF-α and IL-1β, contributing to inflammation and tissue damage. On the other hand, M2 macrophages, stimulated by anti-inflammatory factors, produce immunomodulatory cytokines like IL-10 and TGF-β, promoting tissue healing and resolution of inflammation [12]. An imbalance in macrophage polarization can lead to abnormal immune responses and contribute to the development of various inflammatory diseases. Inflammatory bone resorption, characterized by increased osteoclast activity and bone degradation, is frequently observed in diseases like rheumatoid arthritis and peri-implant osteolysis [13–15]. In scenarios of AL, an inflammatory response contributes to the loss of bone around the prosthesis. Understanding the relationship between macrophage polarization and inflammatory bone resorption/AL is crucial for developing specific treatments to regulate macrophage phenotypes and reduce bone degradation.

In this study, we utilized Ti particles (TiPs) to replicate the impact of wear particles on surrounding tissues by intervening in RAW264.7 cells. Our findings revealed that TiPs effectively enhanced osteoclast activation and bone resorption, while also facilitating the transition of RAW264.7 cells to the M1 pro-inflammatory phenotype. Through RNA sequencing, we identified that TiPs significantly promoted inflammatory response and tumor necrosis factor-activated receptor activity signaling pathways. Among the numerous differential genes, our focus was on the notable upregulation of PTGS2 following the intervention of TiPs. PTGS2, also known as COX-2, is an enzyme involved in the synthesis of prostaglandins and is one of the targets of action of nonsteroidal anti-inflammatory drugs [16, 17]. Researches have also demonstrated that COX-2 was abundantly expressed in the tissue surrounding the failed prosthesis [18]. Previous studies have shown that targeted inhibition of COX-2 to attenuate wear particle-induced inflammatory osteolysis is a viable strategy [19, 20]. Dynastat has been shown to inhibit osteolysis by downregulating the RANK/RANKL signaling pathway in osteoclasts through the inhibition of COX-2 expression [21]. Im *et al.* [19] found that a therapeutic dose of a COX-2 inhibitor (celebrex) might have the potential in inhibiting PPO.

Currently used COX-2 inhibitors, such as celebrex, have been associated with a higher incidence of side effects. For instance, clinical reports have indicated that celebrex can lead to gastrointestinal reactions, including stomach pain, gastric distension and chronic gastritis. Additionally, some patients may experience cardiovascular disorders and allergic reactions [22, 23]. Given the significant impact of traditional Chinese medicine mining on human life sciences and health, our study aimed to identify herbal monomers that have the potential to target COX-2. According to the symmap (www.symmap.org) database, we found that byakangelicol has the potential to target COX-2. Byakangelicol, a natural compound extracted from specific plant sources, has garnered significant interest due to its potential therapeutic properties, particularly in the management of inflammation. Extensive researches have shed light on the pharmacological effects of byakangelicol, highlighting its ability to regulate inflammatory responses [24]. Notably, byakangelicol demonstrates unique anti-inflammatory properties by inhibiting the production of pro-inflammatory cytokines such as TNF-α and IL-1β, while simultaneously enhancing the secretion of anti-inflammatory elements like IL-10 and TGF-β [25]. However, the efficacy of byakangelicol in mitigating wear particle-induced inflammatory bone damage in PPO is yet to be fully explored and warrants further investigation.

In the present study, we evaluated the therapeutic potential of byakangelicol in a classical murine calvarial osteolysis model that emulates particles-induced PPO. We also scrutinized the impact of byakangelicol on the expression of COX-2 and polarization of M1 macrophages instigated by TiPs, both *in vitro* and *in vivo*. Subsequent experiments indicated that byakangelicol might exert its effects by impeding the NF-κB signaling pathway, thereby curtailing osteoclastic differentiation and bone resorption. Additionally, *in vivo* experiments demonstrated that byakangelicol effectively alleviated TiPs-induced inflammatory bone destruction. Our findings imply that byakangelicol exhibits significant potential as a therapeutic modality for treating TiPs-induced osteolysis.

## Materials and methods

### Drugs and reagents

Byakangelicol was purchased from MedChemExpress (New Jersey, USA). TiPs were purchased from Alfa Aesar (Ward Hill, MA). Recombinant RANKL was purchased from R&D Systems (Minneapolis, USA). DMEM/high glucose and fetal bovine serum (FBS) were purchased from Thermo Fisher Scientific (St. Louis, MO, USA). The primary antibodies employed in our experiment included IL-1β (A5871, ABclonal), IL-6 (A0286, ABclonal), TNF-α (A11534, ABclonal), iNOS (A14031, ABclonal), ARG1 (A1847, ABclonal), COX-2 (A3560, ABclonal), NFATc1 (A1539, ABclonal), MMP9 (A0289, ABclonal) and β-Actin (AC006, ABclonal).

### Cell culture

In this study, RAW264.7 cells from the Cell Bank of the Chinese Academy of Sciences (Shanghai, China) were used as osteoclast precursors. We cultured the cells in dulbecco’s modified eagle medium (DMEM)/high glucose supplemented with 10% FBS and antibiotics penicillin/streptomycin every 2–3 days.

### Osteoclast formation assay

After overnight adhesion, RAW264.7 cells were cultured at a density of 2 × 10^4^ cells in a medium containing 50 ng/ml RANKL for 4 days until mature osteoclasts were formed. TRAP staining was performed following the manufacturer’s protocol provided by Suzhou Bizhong Biotechnology Co., Ltd. The cells were fixed with 4% paraformaldehyde before TRAP staining. TRAP-positive osteoblasts (≥3 nuclei) were then observed and photographed using a bright microscope (Zeiss, Germany). ImageJ software (Bethesda, USA) was used to quantify TRAP-positive osteoclasts.

### Processing of TiPs

TiPs were initially treated at 180°C for 12 h and subsequently soaked in ethanol for 2 days to eliminate any remaining endotoxin. The properties of the TiPs were assessed using scanning electron microscopy (SEM). Following previous experiments, we treated RAW264.7 cells with 0.01 mg/ml of TiPs [26]. The morphological features of the cells were observed under an orthogonal microscope (Zeiss, Germany).

### Cytotoxicity assay

5 × 10^3^ RAW264.7 cells were seeded per well in 96-well plates and incubated overnight. Byakangelicol was incubated in RAW264.7 cells at different concentrations (0, 0.01, 0.1, 0.5, 1, 2, 5, 10, 50, 100 μM) for 24 h to assess its toxic potential. Afterward, the Cell Counting Kit-8 (NCM Biotechnology, China) was introduced and incubated for 3 h at 37°C. The cytotoxicity was measured using a microplate reader (BioTek, USA) by detecting the absorbance at 450 nm.

### Immunofluorescence assay

RAW264.7 cells were fixed with 4% paraformaldehyde (Beyotime, China) following various interventions. After permeabilizing with 0.2% Triton X-100 at 4°C, the cells were blocked with QuickBlock buffer (Beyotime, China) for 1 h. We then incubated RAW264.7 overnight at 4°C with the primary antibodies iNOS (A14031, ABclonal) or ARG1(A1847, ABclonal), followed by incubation with FITC-conjugated secondary antibody (Abcam, ab6785) and F-actin (Yeasen, China) for 1 h under dark conditions. In our study on osteoclast skeleton staining, we conducted F-actin ring staining once mature osteoblasts were formed. To begin, the cells were fixed with 4% paraformaldehyde and then stained with F-actin (Yeasen, China) for 1 h. The features of the cells were observed under an orthogonal microscope (Zeiss, Germany).

### Bone resorption assays

The osteoclast function was detected using a bone resorption test. Bovine bone sections (JoyTech Biotechnology, China) were transplanted with RAW264.7 macrophages and induced with RANKL (50 ng/ml) for 8 days. The bone slices were then dehydrated using modified gradient ethanol and dried using the critical point drying method. Gold sputtering of the bone slices was done using an airless jet device. An FEI Quanta 250 SEM (Hillsboro, USA) was used to analyse bone resorption pits.

### Molecular docking

The COX-2 protein was obtained from the PDB database. Subsequently, the 3D structure corresponding to the small molecule was downloaded from PubChem. The optimized small molecule was then imported into AutoDockTools-1.5.6. POCASA 1.1 was employed to predict the protein binding sites. Docking and analysis were performed using AutoDock Vina 1.1.2 and PyMOL 2.3.0, respectively.

### RNA sequencing

RAW264.7 cells were inoculated into six-well plates at a density of 5 × 10^5^. In the intervention group, 0.01 mg/ml of TiPs were used for intervention. TRIzol reagent (Beyotime, China) was used to isolate total RNA after one day of intervention. A sequence of the extracted RNA was performed by GENE DENOVO (Guangzhou, China). Each gene’s FPKM was calculated using Cufflinks. Additionally, reads were obtained for the tested genes. Differential gene expression analysis was performed using the R software package. For the analysis, a *P*-value of 0.05 and a fold change of >1.5 or <0.5 were used as thresholds.

### Western blot

A 30-min lysis was performed on the cell samples using RIPA buffer (Beyotime, China). Protein solution was obtained by centrifuging the supernatant for 20 min at 4°C. Protein quantification was performed using the BCA kit (Beyotime, China). An SDS-PAGE gel was used to separate the samples into equal concentrations, which were then transferred to membranes. Primary antibodies (IL-1β, TNF-α, IL-6, COX-2, MMP9, NFATc1, iNOS, ARG1 and β-actin) were incubated on the membranes at 4°C. The membranes were washed with TBST for 20 min before incubating with secondary antibodies (ab6721, Abcam) for 1 h. We detected antibody–antigen complexes using ECL reagents (NCM Biotech, China). Protein density was quantified using ImageJ software (Bethesda, USA) based on the obtained protein blot images.

### Quantitative real-time PCR

The total RNA of RAW264.7 cells was extracted using TRIzol reagent (Beyotime, China). A NanoDrop 2000 spectrophotometer was used to determine the concentration of RNA. Subsequently, An RNA reverse transcription kit (Thermo Fisher Scientific, USA) was used to convert the RNA into complementary DNA. To perform qPCR amplification, 2 ng of cDNA for each sample was combined with 5 ng of Takara qPCRMasterMix, 0.5 ng each of positive and negative primers and 2 ng of Takara nuclease-free ddH_2_O. The amplification of cDNA was carried out in CFSs using a CFX96™ thermocycler (BioRad Laboratories). Two-dimensional Cq method was used for analysing gene expression. A primer sequence for each target gene can be found in Table 1.

**Table 1.** Primers used in RT-PCR

Gene	Primer sequence (F)	Primer sequence (R)
CTSK	CTTCCAATACGTGCAGCAGA	TCTTCAGGGCTTTCTCGTTC
MMP9	CGTGTCTGGAGATTCGACTTGA	TTGGAAACTCACACGCCAGA
Atp6v0d2	GACCCTGTGGCACTTTTTGT	GTGTTTGAGCTTGGGGAGAA
IL-1β	GAAATGCCACCTTTTGACAGTG	CTGGATGCTCTCATCAGGACA
IL-6	TAGTCCTTCCTACCCCAATTTCC	TTGGTCCTTAGCCACTCCTTC
Ptgs2	TGGAGATCATGGGGAGTCTG	AAGAAAACCTGGTCCGGTGAA
TNF-α	CCCTCACACTCAGATCATCTTCT	GCTACGACGTGGGCTACAG
iNOS	ACATCGACCCGTCCACAGTAT	CAGAGGGGTAGGCTTGTCTC
ARG1	TGGGTGACTCCCTGCATATCT	TTCCATCACCTTGCCAATCC
GAPDH	GGTTGTCTCCTGCGACTTCA	TGGTCCAGGGTTTCTTACTCC

### TiP-induced calvarial osteolysis model

Ethics committee approval was obtained from Soochow University for animal experiments. Twenty-one 8-week-old C57BL/6 male mice were randomly divided into three groups: a sham operation + PBS treatment (sham group), TiPs implantation + PBS treatment (TiPs group) and TiPs implantation + byakangelicol treatment (Bya group). TiPs implantation was shown to calvarial osteolysis. Briefly, the mice were first anaesthetized. Their heads were sterilized and the skin over the skull was cut. Subsequently, a total of 40 µl of PBS solution, with a concentration of 40 mg of TiPs, was carefully administered onto the surface of the mouse skull. In the sham-operated group, a total of 40 μl of PBS was injected and the incision was then sutured. In the Bya treatment group, mice were locally injected with byakangelicol in the skull three times a week until death, with a dose of 1 mg/kg/day. The drug is dissolved in a solution recommended by the reagent manufacturer and injected at a volume of 40 μl per injection. No deaths or complications were observed during the observation period.

### Micro-CT analysis

The mouse calvaria was evaluated using high-resolution micro-CT (SkyScan1176, Belgium) with a resolution of 9 μm, 50 kV and 200 μA. Prior to the evaluations, most of the TiPs were gently removed to prevent metal artifacts in the subsequent analysis. A specific area of interest near the sagittal suture, exposed to TiPs, was examined and three-dimensional images were reconstructed. A variety of morphometric parameters were assessed using SkyScan’s reconstruction program, such as bone mineral density (BMD, mg/cm^3^), bone volume per total volume (BV/TV, %), trabecular number (Tb. N, 1/mm) and number of porosity.

### Histological staining and analysis

A minimum of two days was spent immersing mouse skulls in 10% formalin. Following evaluation using high-resolution micro-CT, the skulls underwent decalcification using 10% ethylenediaminetetraacetic acid (EDTA, Sigma) for a duration of 28 days. Subsequently, the skulls were embedding and slicing into 6-μm sections. These sections were then stained with hematoxylin and eosin staining (H&E), following the experimental procedure used in previous studies, to examine the morphological changes in the tissues [27]. To stain the tissue osteoclasts, we prepared a TRAP staining solution according to the instructions provided by the reagent vendor. The sections were incubated in TARP solution for 30 min and observed under a microscope (Zeiss, Germany). Quantification of the stained osteoclasts was performed using ImageJ software. Immunohistochemistry was performed on 6-µm calvarial sections as per the previously described protocol. Immunofluorescence was conducted by treating them with 4% paraformaldehyde followed by staining. The primary antibodies used were MMP9 (A0289, ABclonal), NFATc1 (A1539, ABclonal), COX-2 (A3560, ABclonal), iNOS (A14031, ABclonal), ARG1 (A1847, ABclonal) and IL-1β (A5871, ABclonal). Quantification of the positive cells was performed using ImageJ software.

### Statistical analysis

All data in our study were presented as mean ± standard deviation and analysed using GraphPad Prism 7.0 software. Statistical significance for multiple comparisons was determined using one-way analysis of variance (ANOVA), and the significance of differences between two groups was verified using Student’s *t*-test. *P* < 0.05 was considered statistically significant.

## Results

### TiPs promoted RANKL-induced osteoclastogenesis and bone resorption *in vitro*

**Figure 1.**
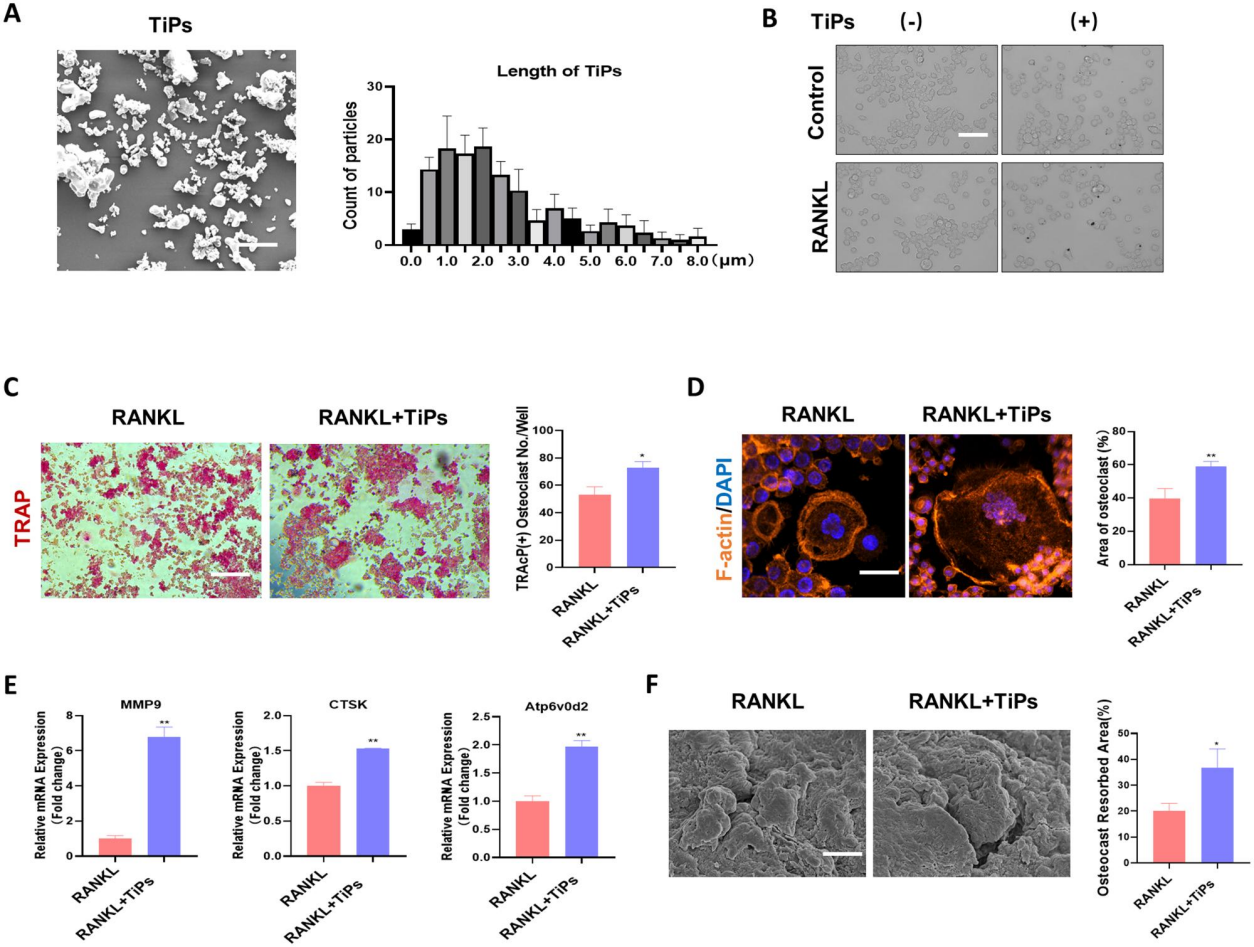
TiPs promoted RANKL-induced osteoclastogenesis and bone resorption *in vitro*. (**A**) Electron micrographs and size analysis of the purchased TiPs. (**B**) Morphological characteristics of RAW264.7 cells after intervention with TiPs and RANKL. (**C**) TRAcP staining and quantitative analysis of the number of positive osteoclasts after intervention with TiPs. (**D**) F-actin staining and quantitative analysis of the area of positive osteoclasts after intervention with TiPs. (**E**) qRT-PCR results showing expression levels of MMP9, CTSK and Atp6v0d2 mRNA. (**F**) Scanning electron microscopy images of the bone resorption area after intervention with TiPs. Scale bar = 50 µm. The data were represented as mean ± SD of 3 independent experiments. *P <* *0.05; **P <* *0.01. qRT-PCR, quantitative real-time PCR.

We utilized scanning electron microscopy to visualize the TiPs and measure their lengths (Fig. 1A). Our analysis demonstrated that the diameter of >90% of the TiPs was under 4 μM. Figure 1B depicts microscopic observations following TiPs introduction. We observed that as time progressed TiPs were gradually phagocytosed into cells. As shown in Fig. 1C, the TRAP staining results indicated a noticeable increase in TRAP-positive cells upon TiPs supplementation. Additionally, immunofluorescent staining of F-actin rings suggested that co-treatment with TiPs amplified osteoclast size (Fig. 1D). In our quest to further understand TiPs’ effect on osteoclastogenesis, we analysed the gene expression of osteoclast-related biomarkers, such as MMP9, CTSK and Atp6v0d2. Our results exhibited a significant elevation of these genes in reaction to TiPs (Fig. 1E). Furthermore, a pit formation assay was carried out to evaluate the influence of TiPs on bone resorption, which demonstrated an enlarged bone resorption area when TiPs were present compared to the RANKL group (Fig. 1E). Collectively, these observations suggest that TiPs potentiate RANKL-induced osteoclastogenesis and bone resorption.

### TiPs facilitated the polarization of macrophages toward the M1 phenotype *in vitro*

**Figure 2.**
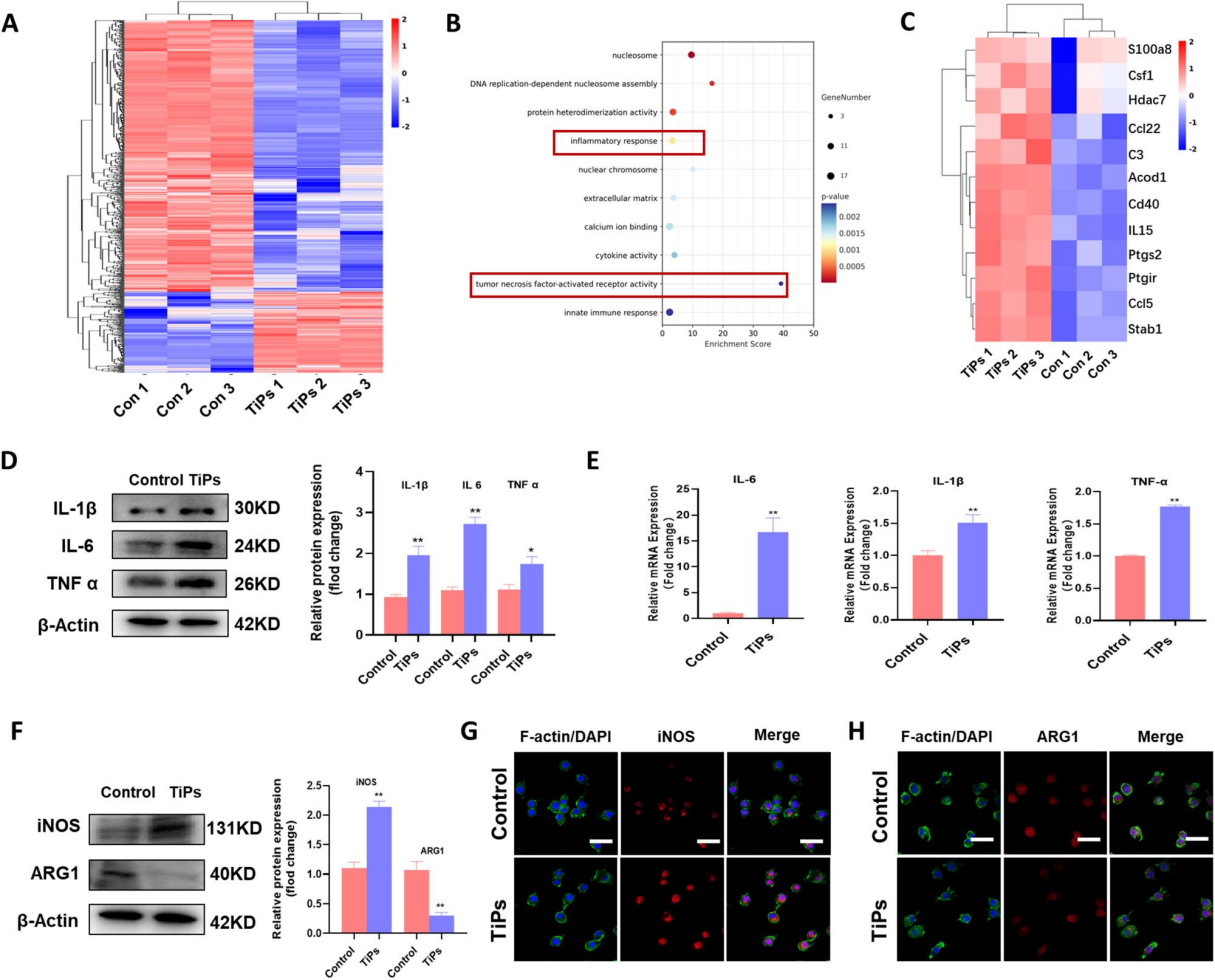
TiPs Facilitated the polarization of macrophages toward the M1 phenotype *in vitro*. (**A**) Heat map showing differentially expressed genes in RAW264.7 in response to TiPs. (**B**) Bubble chart. (**C**) The differences in genes enriched for the inflammatory response and tumor necrosis factor-activated receptor activity pathway. (**D**) Western blot and quantitative analysis showing levels of inflammation-related protein after intervention with TiPs. (**E**) qRT-PCR results showing expression levels of IL-6, IL-1β and TNF-α mRNA. (**F**) Western blot and quantitative analysis showing levels of iNOS and ARG1 after intervention with TiPs. (**G**) Immunofluorescence staining of iNOS and ARG1 in RAW264.7 after intervention with TiPs. Scale bar = 50 µm. The data were represented as mean ± SD of three independent experiments. *P <* *0.05; **P <* *0.01. qRT-PCR, quantitative real-time PCR.

To interrogate the transcriptional alterations in RAW264.7 cells with or without co-treatment with TiPs, we conducted RNA sequencing experiments (Fig. 2A–C). Analysis of the results highlighted a noteworthy surge in the pathway and expression genes of inflammation response and tumor necrosis factor-activated receptor activity. Given the strong link between osteoclastogenesis and pro-inflammatory factors released by M1 macrophages [28], we proceeded to evaluate if TiPs modulated macrophage polarization and consequently influenced osteoclast genesis via the secretion of pro-inflammatory mediators. In line with prior findings, we observed a considerable increase in both protein and mRNA levels of IL-1β, IL-6 and TNF-α after a 24-h intervention with TiPs (Fig. 2D and E). Further, we quantified the levels of iNOS (an M1 macrophage marker) and ARG1 (an M2 macrophage marker) employing Western blotting and immunofluorescence techniques. As illustrated in Fig. 2F and G, we found a pronounced enhancement in the expression of iNOS, while the expression of ARG1 was notably diminished in the presence of TiPs. In conclusion, these data propose that TiPs can stimulate a shift in macrophage phenotypic transition, favoring the M1 phenotype, and elevating the production of pro-inflammatory cytokines.

### Byakangelicol inhibited osteoclastogenesis and bone resorption by suppressing the TiPs-induced upregulation of COX-2 *in vitro*

**Figure 3.**
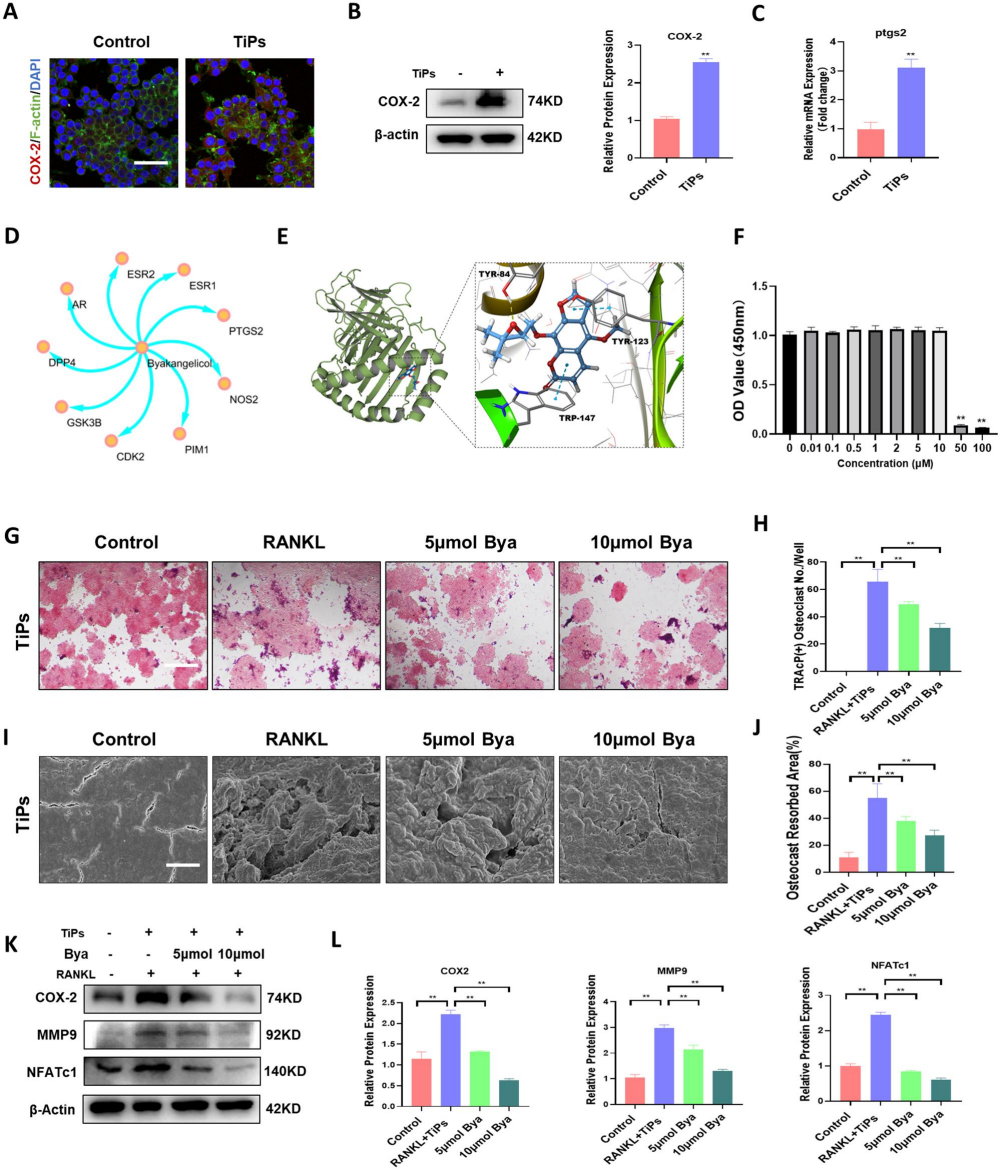
Byakangelicol inhibited osteoclastogenesis and bone resorption by suppressing the TiPs-induced upregulation of COX-2 *in vitro*. (**A**) Immunofluorescence staining of COX-2 in RAW264.7 after intervention with TiPs. (**B**) Western blot and quantitative analysis showing levels of COX-2 after intervention with TiPs. (**C**) qRT-PCR results showing expression levels of COX-2 mRNA (ptgs2) after intervention with TiPs. (**D**) Potential targets of action for byakangelicol. (**E**) Molecular docking. (**F**) CCK-8 assay of byakangelicol, *n* = 5. (**G** and **H**) Representative TRAP staining images and quantification of TRAP-positive cells. (**I** and **J**) SEM images and quantification of osteoclast resorbed area of the bone resorption after byakangelicol treatment. (**K** and **L**) Western blot and quantitative analysis showing levels of COX-2, MMP9 and NFATc1 after intervention with TiPs. Scale bar = 50 µm. The data were represented as mean ± SD of three independent experiments. *P <* *0.05; **P <* *0.01. qRT-PCR, quantitative real-time PCR.

As shown in Fig. 2C, we screen for inflammation-related differential genes among the entire set of differential genes in RAW264.7 cells following TiPs intervention. Analysis of the results highlighted a surge in the expression of inflammation-related genes, specifically ptgs2 (the gene coding for COX-2), a gene deeply intertwined with inflammation and macrophage polarization processes. Previous studies have demonstrated a noteworthy increase in patient tissue COX-2 expression during the progression of PPO [29]. In our study, immunofluorescence data demonstrated that TiPs could significantly enhance the expression of COX-2 in macrophages (Fig. 3A). This was further corroborated by Western blot and qPCR results, which indicated an upregulation of COX-2 expression in the presence of TiPs (Fig. 3B and C). These findings suggest that TiPs are capable of promoting the upregulation of COX-2 in macrophages, supporting our earlier research that inhibiting COX-2 could mitigate TiPs-induced bone resorption. Given the adverse effects associated with current COX-2 inhibitors, such as exacerbation of chronic kidney disease, gastrointestinal complications and increased cardiovascular risk, we sought a more suitable pharmaceutical that could both inhibit COX-2 and be employed in the treatment of wear debris-induced osteolysis. With the rapid development of traditional Chinese medicine and the potential revealed through the modern exploration of traditional drugs, we screened for byakangelicol from a Chinese medicinal database (www.symmap.org), a COX-2 inhibitor, and simulated its action site (Fig. 3D). As shown in Fig. 3E, hydrogen bonds were observed between TYR-84 of COX-2 and byakangelicol (indicated by a yellow dashed line). Additionally, Pi-Pi stacking interactions were formed between TYR-123 and TRP-147 of COX-2 with byakangelicol (indicated by a blue dashed line). The Glide-score is calculated to be -9.6353, indicating a strong interaction between the byakangelicol and COX-2. Cytotoxicity assays indicated that concentrations of byakangelicol below 10 μM had no significant impact on macrophage proliferation (Fig. 3F). We proceeded to investigate byakangelicol’s capacity to influence osteoclast differentiation by treating RAW264.7 cells with RANKL and TiPs, and a range of byakangelicol concentrations, demonstrating that byakangelicol obstructs osteoclast formation in a concentration-dependent fashion (Fig. 3G and H). In line with these outcomes, we employed a pit formation assay and discovered that byakangelicol reduced the bone resorption area in a concentration-dependent manner (Fig. 3I and J). Western blot and qPCR confirmed the upregulation of osteoclastic markers MMP-9 and NFATc1 by TiPs. Upon the addition of byakangelicol, the upregulation of COX-2 and the increase of MMP-9 and NFATc1 were inhibited. These results suggest that byakangelicol may ameliorate TiPs-stimulated COX-2 upregulation, thus reducing osteoclast differentiation and bone resorption.

### Byakangelicol inhibited TiP-induced polarization of macrophages toward the M1 phenotype *in vitro*

**Figure 4.**
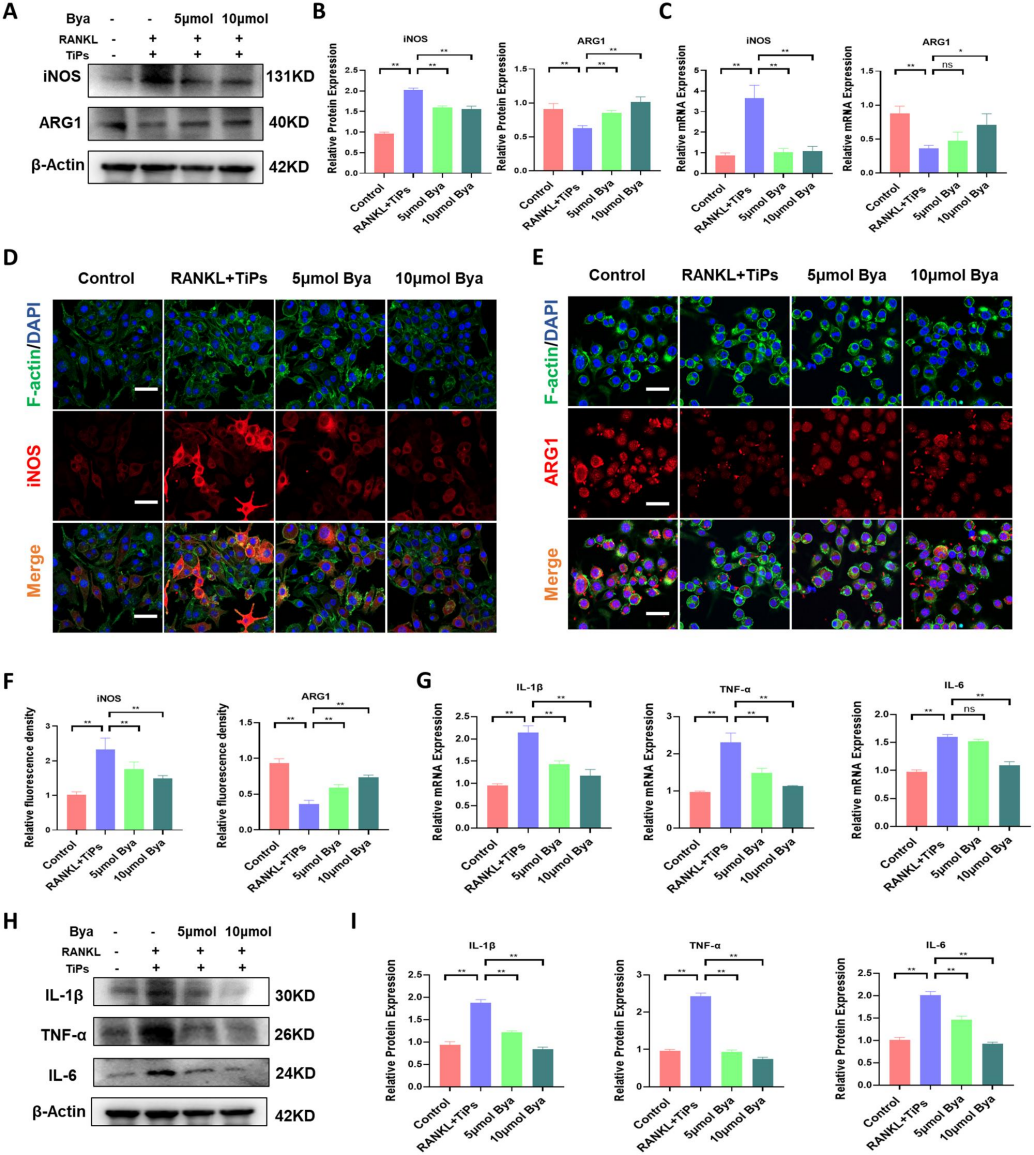
Byakangelicol inhibited TiP-induced polarization of macrophages toward the M1 phenotype *in vitro*. (**A** and **B**) Western blot and quantitative analysis showing levels of iNOS and ARG1 after 24 h intervention with TiPs and RANKL. (**C**) RT-PCR analysis of the mRNA expression levels of iNOS and ARG1. (**D–F**) Representative images and quantitative analysis of immunofluorescence staining; red (iNOS and ARG1), green (F-actin) and blue (nuclei). Scale bar = 50 µm. (**G**) RT-PCR analysis of the mRNA expression levels of IL-1β, TNF-α and IL-6 after intervention with TiPs and RANKL. (**H** and **I**) Western blot and quantitative analysis showing levels of IL-1β, TNF-α and IL-6 after intervention with TiPs and RANKL. Scale bar = 50 µm. The data were represented as mean ± SD of 3 independent experiments. *P <* *0.05; **P <* *0.01.

As demonstrated in Fig. 4A and B, byakangelicol effectively attenuated the surge in iNOS expression caused by TiPs, while simultaneously boosting the ARG1 levels. These observations were substantiated by qPCR analysis results (Fig. 4C). Further, we probed into the expression dynamics of iNOS and ARG1 using immunofluorescence assays. The data revealed a significant augmentation in iNOS expression triggered by TiPs, which was mitigated upon the administration of byakangelicol. Concurrently, the ARG1 expression, dampened by TiPs, was observably amplified in the presence of byakangelicol (Fig. 4D and E). Moreover, we examined the influence of byakangelicol on the manifestation of pro-inflammatory factors. As illustrated in Fig. 4F–H, TiPs considerably elevated the expression of IL-1β, TNF-α and IL-6 at both mRNA and protein levels. However, this enhancement was substantially diminished upon the introduction of byakangelicol. Taken together, these results demonstrated the potential of byakangelicol to temper TiPs-elicited pro-inflammatory cytokine release and impede the shift toward the M1 macrophage phenotype.

### Byakangelicol suppressed the COX-2/NF-κB signaling pathway *in vitro*

**Figure 5.**
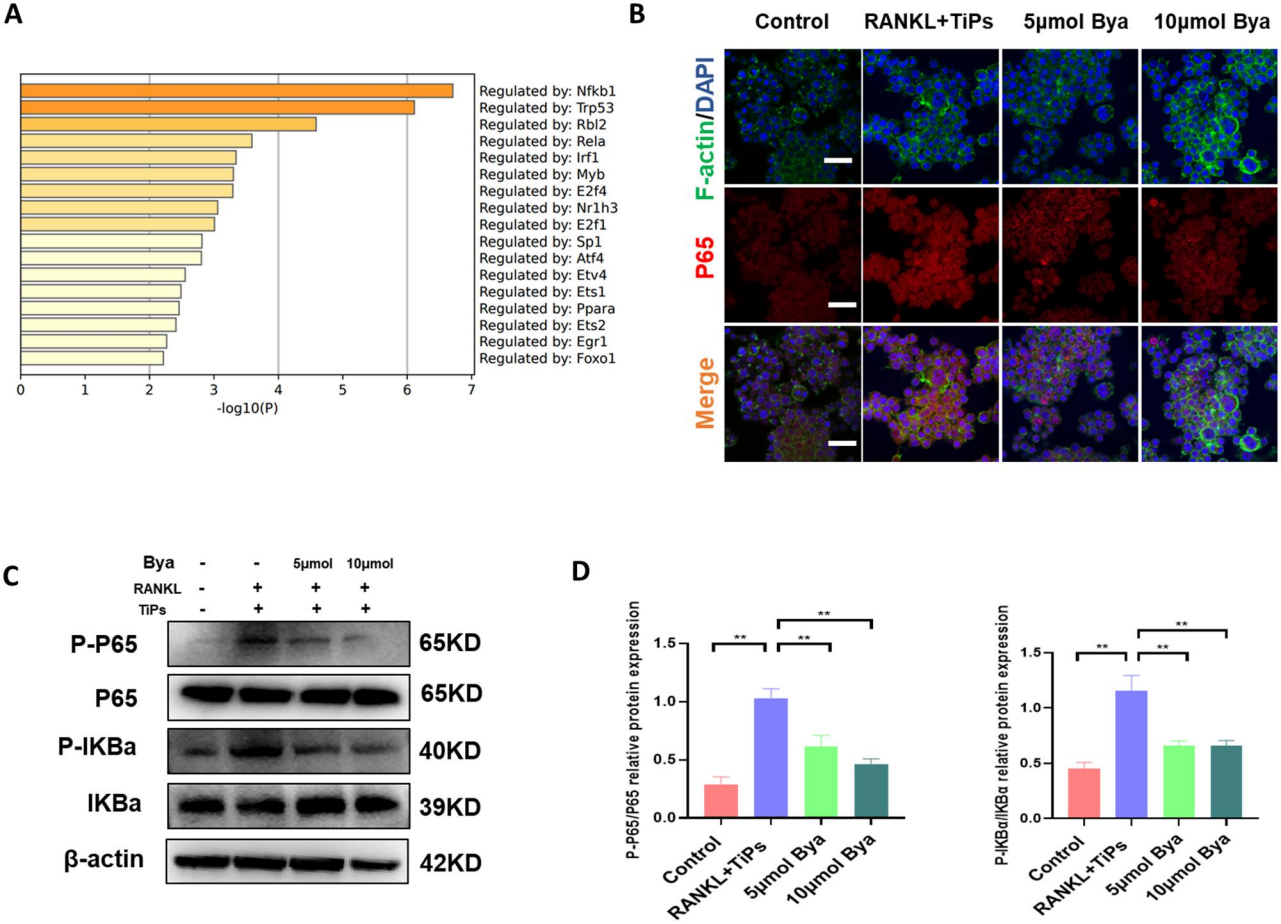
Byakangelicol suppressed the COX-2/NF-κB signaling pathway *in vitro*. (**A**) GO enrichment bar graph analysis. (**B**) Immunofluorescence staining of P65 in RAW264.7 after intervention with TiPs and RANKL. (**C** and **D**) Western blot showing levels of P-P65, P65, P-IKBα and IKBα and after intervention with byakangelicol and quantitative analysis of P-P65/p65 and P-IKBα/IKBα. The data were represented as mean ± SD of three independent experiments. *P <* *0.05; **P <* *0.01.

Insights gleaned from our RNA sequencing experiments revealed the potential of TiPs to boost the upregulation of NF-κB (Fig. 5A). As prior research has underscored the pivotal role of the COX-2/NF-κB signaling system in the guidance of macrophage polarization toward the M1 phenotype and in the production of pro-inflammatory cytokines, we posited that byakangelicol might orchestrate macrophage phenotype switch and cytokine release by inhibiting the COX-2/NF-kB signaling axis [30]. To test this hypothesis, we subjected TiPs-stimulated macrophages to byakangelicol treatment. As our immunofluorescence data illustrated, byakangelicol exerted a concentration-dependent blockade of the TiPs-induced elevation of P65, a fundamental factor in the NF-κB signaling system (Fig. 5B). In alignment with this observation, western blot results indicated a dose-related decrease in the production of P-IKBα and P-P65, signifying the byakangelicol-triggered repression of the NF-κB pathway (Fig. 5C and D). These outcomes intimated that byakangelicol could mediate the redirection of macrophages toward the M1 phenotype and the control of pro-inflammatory cytokine secretion through its interaction with the COX-2/NF-κB signaling pathway.

### Byakangelicol alleviated TiPs-triggered bone resorption in a mouse calvarial osteolysis model

**Figure 6.**
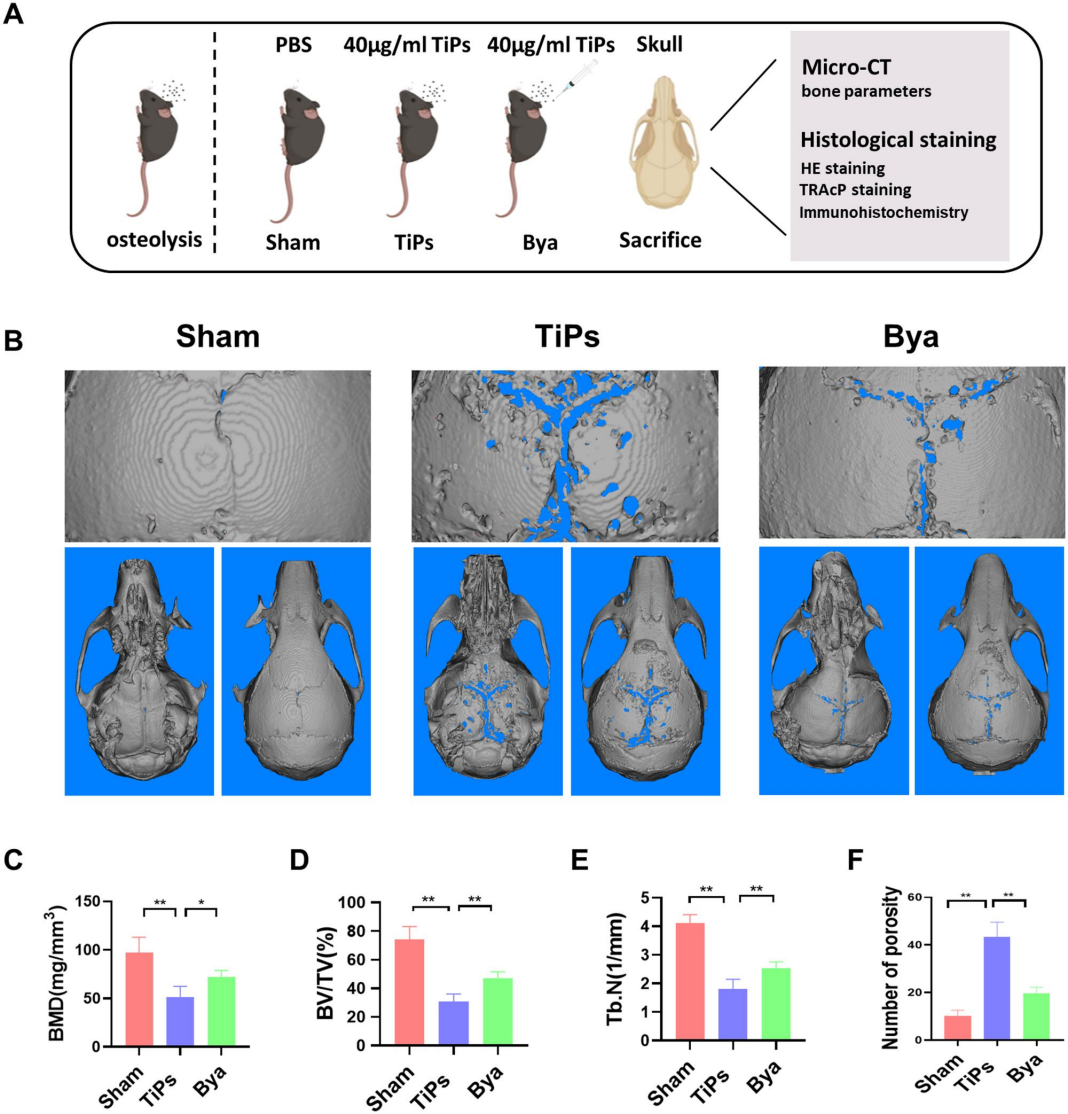
Byakangelicol alleviated TiP-triggered bone resorption in a mouse calvarial osteolysis model. (**A**) Schematic diagram of mice intervention. (**B**) Representative 3D and 2D reconstruction images of micro-CT. (**C**–**F**) Related bone parameters. *n* = 7 per group. The data were represented as mean ± SD. *P <* *0.05, **P <* *0.01.

Figure 6A depicts a flow chart representing the entire animal operation. As depicted in Fig. 6B, TiPs led to a significant augmentation in bone absorption in mouse calvariae, which was partially counteracted by byakangelicol. The quantitative evaluation revealed that TiPs profoundly diminished BMD, BV/TV and Tb. N while concurrently escalating the count of lytic pores in the mice calvariae. Importantly, byakangelicol administration displayed a marked suppression of TiPs-elicited bone resorption compared to the sham group (Fig. 6B–F).

**Figure 7.**
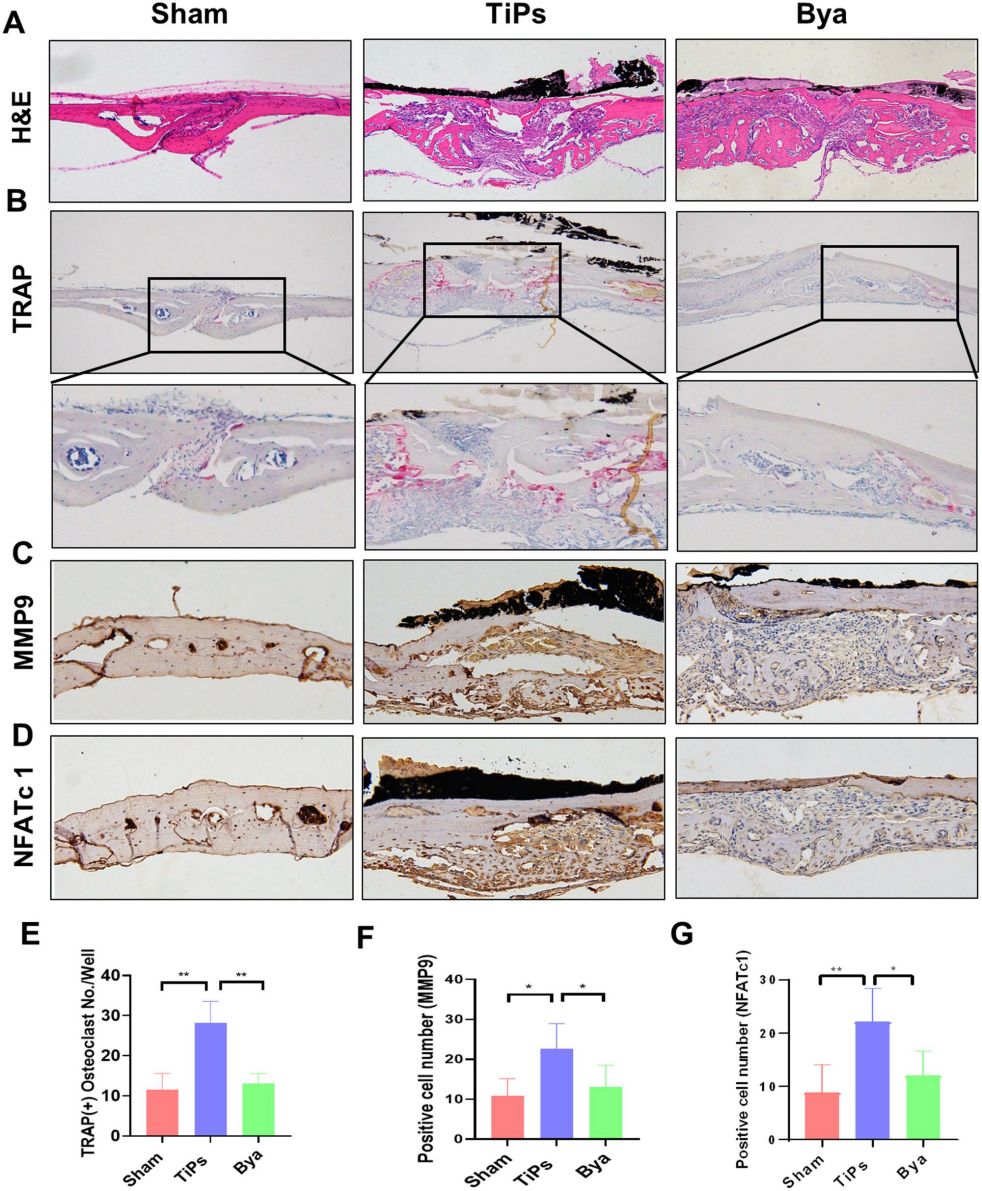
Byakangelicol alleviated osteoclasts’ formation in a mouse calvarial osteolysis model. (**A**) H&E staining of cranial tissues of mice in various groups. (**B** and **E**) TRAP staining and quantitative analysis of osteoclasts in cranial tissues of mice. (**C** and **F**) Immunohistochemical staining and quantitative analysis for MMP9 of decalcified bone sections. (**D** and **G**) Immunohistochemical staining and quantitative analysis for NFATc1 of decalcified bone sections. *n* = 7 per group. The data were represented as mean ± SD. *P <* *0.05, **P <* *0.01.

Further evidence supporting byakangelicol’s ameliorative effect on particle-instigated bone resorption *in vivo* came from histological and histomorphometric analyses of calvarial samples. H&E staining manifested an enhancement in erosion in the TiPs group relative to the sham group, which was significantly attenuated by byakangelicol (Fig. 7A). Intensive TRAP-positive staining was observed in the zones of implanted calvariae and resorptive lacunae, sites of active osteolysis. As indicated in Fig. 7B and E, the number of mature osteoclasts remarkably escalated in response to TiPs, whereas a nearly 50% reduction in the osteoclast number was recorded in the group treated with byakangelicol. Consistent with these findings, Fig. 7C and D and F and G exhibits an increase in osteoclast-related marker genes MMP9- and NFATc1-positive cells in the calvariae of the TiPs group when compared with the sham group, while the byakangelicol-treated groups exhibited diminished expression levels of MMP9- and NFATc1-positive cells. These outcomes imply that byakangelicol might mitigate TiPs-induced bone resorption *in vivo* via the reduction of osteoclasts.

### Byakangelicol inhibited the polarization of macrophages toward the M1 phenotype *in vivo*

**Figure 8.**
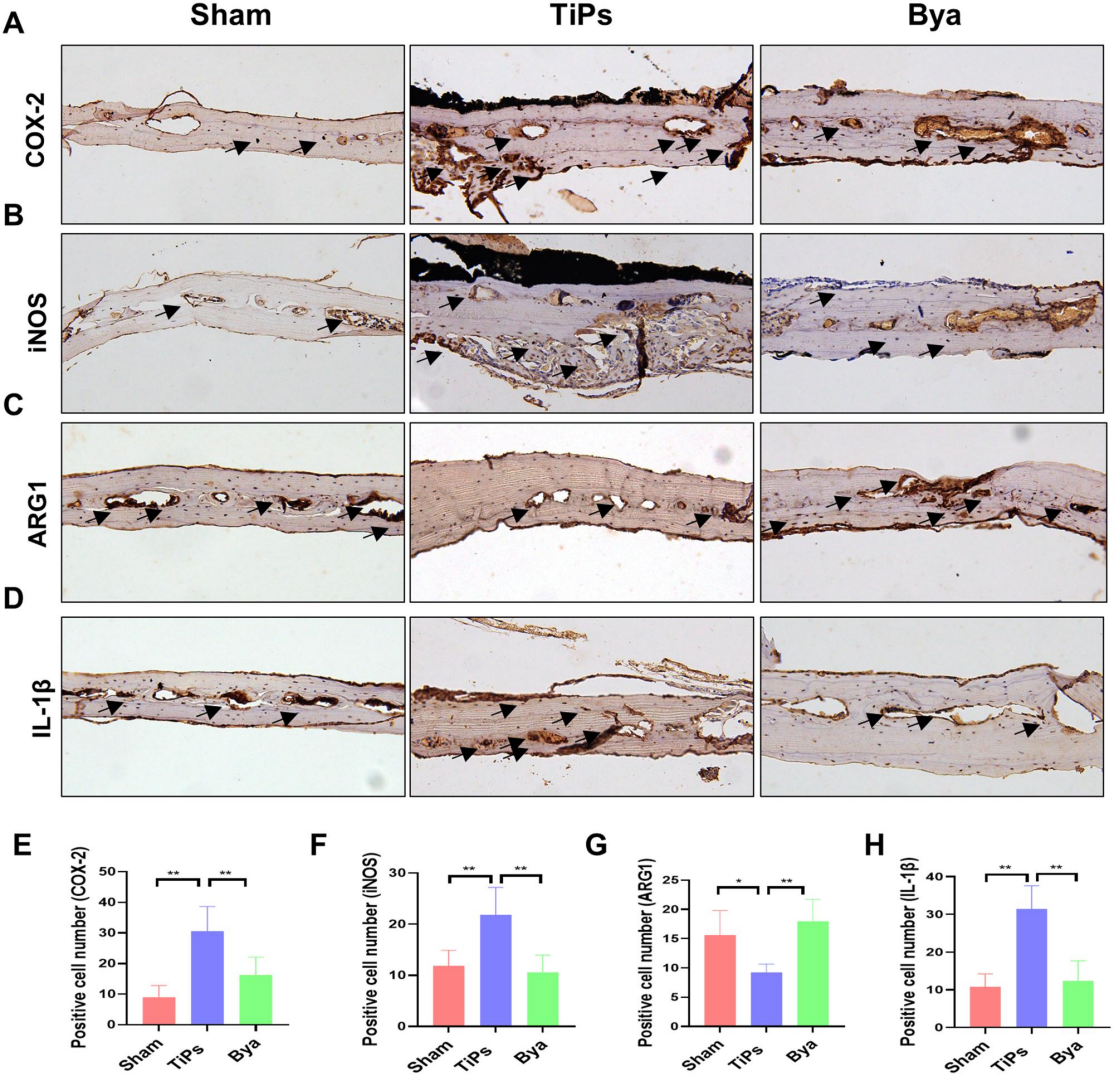
Byakangelicol inhibited the polarization of macrophages toward the M1 phenotype *in vivo*. (**A** and **E**) Immunohistochemical staining and quantitative analysis for COX-2 of decalcified bone sections. (**B** and **F**) Immunohistochemical staining and quantitative analysis for iNOS of decalcified bone sections. (**C** and **G**) Immunohistochemical staining and quantitative analysis for ARG1 of decalcified bone sections. (**D** and **H**) Immunohistochemical staining and quantitative analysis for IL-1β of decalcified bone sections. *n* = 7 per group. The data were represented as mean ± SD. *P <* *0.05, **P <* *0.01.

Figure 8A–H reveals that TiPs amplified the expression of COX-2 within mouse calvariae while concurrently elevating the quantities of M1 macrophage indicators iNOS and IL-1β and diminishing the level of the M2 macrophage indicator ARG1. When the mice were treated with byakangelicol, there was a marked reduction in COX-2 expression, a substantial suppression of the increased levels of iNOS and IL-1β and a reversal of the decreased ARG1 level. These findings indicate that byakangelicol may inhibit COX-2 *in vivo*, which in turn mitigates the TiPs-induced shift of macrophages toward the M1 phenotype.

## Discussion

AL represents a significant challenge in the field of orthopedics following total joint arthroplasty [31, 32]. This condition has been identified as a leading cause of failure for implanted prosthetics, prompting the need for repeated surgical interventions. Central to the development of AL is the role of macrophages [33]. Once activated by wear particles from the prosthetic material, macrophages orchestrate an inflammatory response characterized by the secretion of pro-inflammatory cytokines. These cytokines primarily secreted by M1-type macrophages, are implicated in promoting osteoclastogenesis and subsequently bone resorption [34]. Thus, therapeutic strategies targeting these inflammatory processes, particularly the suppression of M1 macrophages, could provide an effective method to manage and potentially prevent AL, improving outcomes for patients undergoing joint arthroplasty.

The progress in research on mitigating wear particle-induced osteolysis by suppressing M1 macrophages has been substantial [35, 36]. It has been observed that inhibiting the activity of M1 macrophages can decrease the production of pro-inflammatory cytokines that exacerbate osteolysis. Gao, et al. found that NF-κB participated in the PMMA-induced M1 macrophage polarization, specifically through the involvement of let-7f-5p [37]. Pajarinen *et al.* reported that IL-4 treatments were found to be highly effective in preventing and repairing preexisting bone loss induced by polyethylene. This was achieved by reducing the number of iNOS^+^ M1 macrophage and increasing the number of CD206^+^ M2 macrophages [38]. Moreover, their studies showed that IL-4-treated reduced the number of osteoclasts. Consequently, there is been a shift in the focus toward exploring ways to modulate macrophage polarization from the M1 phenotype toward the M2 phenotype. Studies are investigating pharmacological interventions that could inhibit M1 macrophages and limit wear particle-induced osteolysis [39]. Zhao *et al.* [40] found that ultra-high molecular weight polyethylene particles induced the expression of pro-inflammatory cytokines TNF-α and IL-6, while metformin inhibited these variations and promoted the release of anti-inflammatory cytokine IL-10. To examine the influence of TiPs on osteoclast differentiation, we introduced TiPs to RAW264.7 cells which were stimulated with 50 ng/ml RANKL and monitored the ensuing osteoclast differentiation. In the present study, we observed that TiPs promoted the polarization of macrophages toward the pro-inflammatory M1 phenotype, characterized by increased expression of ptgs2 (the gene coding for COX-2), IL-1β, IL-6, TNF-α and iNOS. TiPs further induced a remarkable decrease in the expression of ARG1, a marker associated with the anti-inflammatory M2 phenotype. This transition suggests that TiPs could induce a pro-inflammatory state that may exacerbate osteoclastogenesis and bone resorption. Upon using byakangelico to inhibit COX-2, we observed a significant decrease in pro-inflammatory cytokines as well as M1 macrophage-related markers, such as iNOS. Importantly, byakangelico could notably inhibit osteoclast-induced bone resorption in both *in vitro* and *in vivo* experiments.

Byakangelicol has been gaining increasing attention for its relation to inflammation [25]. As a naturally occurring compound found in certain plants, byakangelicol has been demonstrated to have potent anti-inflammatory properties. It has demonstrated its potential in mitigating the inflammatory response by inhibiting COX-2, a key enzyme involved in the generation of pro-inflammatory mediators. By acting on COX-2, byakangelicol can reduce the release of pro-inflammatory cytokines, which are proteins that regulate the immune response and inflammation [24]. Our analysis demonstrated that byakangelicol was capable of counteracting the effects of TiPs in inducing osteoclastogenesis, as evident by its ability to diminish the expression of osteoclastogenic markers such as MMP-9 and NFATc1.

To ascertain the effect of byakangelicol on the TiPs-stimulated transition of macrophages toward the pro-inflammatory M1 phenotype, we exposed TiPs-activated macrophages to different concentrations of byakangelicol. Further *in vitro* experiments found that byakangelicol inhibited the TiPs-induced polarization of macrophages toward the M1 phenotype, as demonstrated by the reduction in iNOS expression and concurrent increase in ARG1 levels, thereby indicating its potential to hinder the pro-inflammatory response and osteolysis associated with TiPs exposure. Furthermore, our findings revealed that byakangelicol exerts its inhibitory effects by suppressing the COX-2/NF-κB signaling pathway. Byakangelicol showed a concentration-dependent decrease in the expression of P-IKBα and P-P65, key players in the NF-κB signaling cascade. This suppression highlights the potential of byakangelicol to mediate the M1 macrophage phenotype switch and control the pro-inflammatory cytokine secretion, thereby presenting a promising therapeutic strategy.

To investigate the inhibitory action of byakangelicol on bone resorption *in vivo*, we adopted a mouse model of calvarial osteolysis and employed micro-CT along with histological staining for the assessment. *In vivo* experiments in a mouse calvarial osteolysis model further supported the *in vitro* findings. Byakangelicol administration led to a substantial reduction in TiPs-induced bone resorption, TRAP-positive staining and osteoclast-related gene expression, reinforcing the notion that byakangelicol can mitigate TiPs-induced bone resorption *in vivo* via the reduction of osteoclasts. In an endeavor to discern the influence of byakangelicol on *in vivo* macrophage phenotypes, we conducted histological and histomorphometric evaluations in mouse calvariae. Moreover, histological evaluations corroborated the capacity of byakangelicol to inhibit the TiPs-induced polarization of macrophages toward the M1 phenotype. The specific efficacy of byakangelicol in clinical use has not been determined yet due to the numerous challenges encountered in translating basic research into clinical strategies. However, byakangelicol possesses a combination of analgesic, antipathogenic microbial, antitumor, hepatoprotective and various other pharmacological activities [41–43]. Therefore, it is possible that byakangelicol could represent a significant advancement in addressing PPO.

While our research presents some compelling results, we acknowledge several limitations that warrant further exploration. First, we used TiPs as an osteolysis model instead of the more clinically relevant ultra-high molecular weight polyethylene debris, typically found in patients experiencing peri-implant failures. Our choice of TiPs is premised on the consideration that various wear particles share fundamental similarities in their elicited biological responses, including inflammation and the promotion of osteoclastogenesis. These similarities have been robustly confirmed by a plethora of experimental evidence. Second, the administration of byakangelicol was limited to a two-week duration, which may not accurately represent the timeline of clinical treatment. Therefore, future research could benefit from evaluating the long-term effects and safety profile of byakangelicol over an extended period. Moreover, the use of a calvarial model may not offer a perfect simulation of clinical PPO. The model involves a single injection of TiPs onto the calvarial surface, diverging from the continuous particle release that occurs at the bone-implant interface in a clinical setting. Lastly, we did not incorporate aspects such as fluid pressure or mechanical load during this process, which are factors present in patients and could potentially influence the results. Hence, further research will need to account for these aspects to provide a more comprehensive understanding of the influence of byakangelicol on osteolysis.

## Conclusions

In summary, our study provides new insights of TiPs in osteoclastogenesis and bone resorption, and the potential of byakangelicol in mitigating these adverse effects. By inhibiting the COX-2/NF-κB signaling pathway and restraining macrophage polarization toward the M1 phenotype, byakangelicol offers a promising therapeutic strategy for managing wear debris-induced osteolysis. Future studies are warranted to further investigate the molecular mechanisms involved and to explore the clinical applicability of byakangelicol in treating wear debris-induced osteolysis.

## Data Availability

The datasets generated and/or analysed during the current study are not publicly available but are available from the corresponding author on reasonable request.
